# Cortical and subcortical changes following sphenopalatine ganglion blocks in chronic migraine with medication overuse headache: a preliminary longitudinal study

**DOI:** 10.1186/s40695-020-00055-y

**Published:** 2020-08-05

**Authors:** Roger D. Newman-Norlund, Chris Rorden, Nasim Maleki, Milap Patel, Brian Cheng, X. Michelle Androulakis

**Affiliations:** 1 Department of Psychology, University of South Carolina, 915 Greene Street, Discovery I Building, Office 138, Columbia, SC 29208 USA; 2 Harvard Medical School, Boston, MA USA; 3 Department of Neurology, University of South Carolina, Columbia, SC USA; 4 Division of Neurology, WJB Dorn VA Medical Center, Columbia, SC USA

**Keywords:** Chronic migraine, Medication overuse headache, Sphenopalatine ganglion, Voxel-based morphometry, Cortical thickness

## Abstract

**Objective:**

The purpose of this pilot study was to investigate potential changes in brain morphology (cortical thickness and cortical/subcortical volume) accompanying a series of sphenopalatine ganglion (SPG) blockade treatments in chronic migraine with medication overuse headaches (CM^w/MOH^).

**Background:**

Local anesthetization of the SPG via intranasal application is used for the treatment for multiple types of headache disorders, including CM. Our previous longitudinal fMRI study revealed improved network connectivity after such treatment. However, the impact of SPG blocks on cortical, subcortical gray matter volume and cortical thickness has yet to be assessed.

**Methods:**

Using magnetic resonance imaging (MRI), cortical/subcortical volume were measured in 12 chronic migraine patients before and after a series of 12 SPG blocks administered over a 6-week period (2 per week). The average time between MRI assessments was 6 weeks. Targeted, within-subjects t-tests comparing pre-treatment and post-treatment values in specific apriori brain regions of interest, including the hippocampus, amygdala, basal ganglia, somatosensory cortex, temporal cortex and occipital cortex, were used to estimate the impact of repetitive SPG blocks treatment on brain morphology in CM^w/MOH^.

**Results:**

Compared to baseline values, the number of moderate/severe headache days per month, HIT-6, PHQ-9 scores and allodynia scores were all significantly improved at the end of treatment. Analysis of MRI data revealed that the volume of the right hippocampus and the right palladium significantly decreased following SPG block treatment, while the volume of the left nucleus accumbens significantly increased following treatment. Cortical thickness in the left temporal pole and left lateral occipito-temporal gyrus significantly decreased following SPG block treatment.

**Conclusion:**

Our results suggest SPG block treatment is associated with significant symptom improvement as well as significant structural brain changes in regions known to be associated with migraine and chronic pain processing in CM^w/MOH^.

## Background

The sphenopalatine ganglion (SPG) has been implicated in cephalalgia for over a century. It is now widely known that disruption of neural signals generated by the SPG can modulate the output of the autonomic nerve fibers involved in headache. This disruption can be induced via electrical stimulation, or as is more commonly the case, through the administration of local anesthetics to the areas in close vicinity of SPG [1–7]. While some of the currently available approaches are either invasive (involving penetration of the mucosa on the lateral nasal wall) or expensive (requiring fluoroscopic guidance), SPG block using local anesthesia is a novel treatment option with great clinical potential and is both non-invasive and relatively inexpensive [8]. However, questions remain concerning the possible effects of this treatment on brain structures known to modulate migraine pain. Subcortically, these structures include the hippocampus [9–12], amygdala [13–15] and basal ganglia [9, 11, 16]. Regarding the hippocampus and amygdala, studies suggest that functional activity, connectivity with other regions, and volume are modulated by migraine status [9–15]. Specific components of the basal ganglia, namely the caudate, putamen, palladium and nucleus accumbens have additionally been implicated in migraine [11, 16]. A significant body of literature also supports a relationship between migraine and various cortical components. There appears to be a strong link between migraine status and structure of the somatosensory cortex, with multiple studies reporting a thickening of S1 in migraineurs [17, 18]. While evidence for a relationship between migraine and brain structure is less strong in other areas, there is reasonable evidence that two additional sites are involved in the migraine brain. Schwedt and colleagues reported increased cortical thickness the temporal lobe in participants with migraine [19] and structural abnormalities have been reported in visual areas in migraineurs [20].

While several studies hint at potential relationships between brain structures and various aspects of migraine (such as severity and frequency) [9–20], little is known about the potential effects of SPG treatment on brain structure and function. Recently, our lab conducted a pilot longitudinal fMRI study on the use of repetitive SPG blockades for chronic migraine with medication overuse headache (CM^w/MOH)^, which demonstrated improved overall central executive network connectivity and intra-network connectivity within salience network [21]. Here, we evaluated whether this treatment is associated with any changes in brain morphometry. Specifically, we examined the effect of SPG blockade on brain volumetric changes in cortical and subcortical regions, pre and post treatment in women with CM^w/MOH^. Based on prior literature [9–20], we identified our regions of interest as following: amygdala, hippocampus, basal ganglia, somatosensory cortex, temporal cortex and occipital cortex.

## Materials and methods

### Participants

Participants were eligible for the study if they were 18 years or older and met ICHD III beta diagnostic criteria for CM with MOH, as determined by a headache specialist. All participants had predominantly frontal and/or orbital pain​. All participants agreed not to start new migraine preventive medications or take a stable dose of preventative medications for at least 60 days prior to enrolling to the study. Patients were scanned at their baseline level of pain and at least 24 h outside of their acute pain exacerbation period; any patient who came in within 24 h of acute pain exacerbation was rescheduled. Additionally, participants were required to meet all requirements specified by the device manufacturer.

Exclusion criteria included cervicogenic headaches, or other headache treatment procedures such as nerve blocks or onabotulinumtoxinA (Botox), physical therapy, or acupuncture 6 months before or during the treatment period. Exclusion criteria also included MRI contraindications such as presence of non-removable metal or metal containing devices. Participants were excluded if they had neurological or pain disorders other than CM^w/MOH^, or chronic illness (i.e., hypertension, diabetes, hepatic, renal, chronic inflammatory, or infectious disease, etc.). Participants were also excluded if they had any chronic illness that could have impact their adherence to the treatment program (i.e. hypertension, diabetes, hepatic, renal, chronic inflammatory or infectious disease). Thirteen participants were initially enrolled into the study. One participant had to be excluded due to failure to complete the treatment phase, thus leaving a total of twelve patients for the final analysis. All of the 12 remaining patients completed the 6-week treatment course and were imaged at two time points. Eight out of the 12 participants completing the experiment reported receiving migraine prophylaxis. Patients reported using nifedipine (*N* = 1), metoprolol (N = 1), amitriptyline (N = 1), topiramate (*N* = 5). Some patients in this cohort also participated a previously published functional MRI study in which we examined 10 individuals with CM^w/MOH^ [21].

During the 6-week treatment phase, participants were instructed to continue with the prophylactic medications, as needed. The adoption of new medications or treatments was not permitted during the treatment period. Each participant was scanned (1) immediately before their first SPG treatment, and (2) 30 min after the last treatment of a 6-week regimen consisting of a series of 12 SPG blocks administered twice per week. Participants were scanned at their baseline level of pain; any participant who came in within 24 h of acute pain exacerbation was rescheduled.

### Clinical parameters

Standard neurological examinations were conducted for all participants, and vital signs were measured prior to each MRI session. Standardized questionnaires were used to ascertain clinical characteristics and demographic information (i.e., age, sex, race, BMI, and educational level). A number of relevant clinical characteristics were recorded including: (1) duration of migraine history, (2) duration of CM history, (3) family history of migraine, (4) current medications, (5) number of moderate to severe headache days per month, (6) location of migraine, (7) presence of aura, (8) headache-related disability as determined by Headache Impact Test (HIT-6VR), [22] (9) depression as determined by Patient Health Questionnaire (PHQ-9), [22, 23] (10) allodynia as measured by allodynia symptom checklist (ASC 12) [24].

### Standard protocol approvals, registrations, and participant consent

The study protocol was approved by the institutional review board at University of South Carolina. Written informed consent was obtained from all participants.

### Transnasal SPG block

In this study, all CM^w/MOH^ participants received a series of 12 SPG blockade (twice per week for 6 weeks) using the Tx360VR (Tian Medical Inc.; Lombard, IL, USA) with 0.5% bupivacaine. This device uses a small, flexible, soft plastic catheter to advance below the middle turbinate just past the sphenopalatine foramen. The plastic tube can then be rotated laterally on a preset track and extended into the intranasal cavity. A total of 0.3 mL of 0.5% bupivacaine is administered into each nostril over the mucosa covering the SPG [8, 25]. Dosing and anesthetic type was determined per device manufacturer’s recommendations.

### MR imaging

All participants were scanned on a Siemens 3 T scanner located at the McCausland Center for Brain Imaging (Columbia, South Carolina). This system was upgraded from a Trio to a Prisma configuration during this study. Each individual was only scanned with one of these configurations prior to and after SPG treatment (no single participant was scanned on two different MR systems), and the configuration was included as a nuisance parameter in our analyses. A T1-weighted MP-RAGE scan was acquired both prior to and after the treatment phase of the study. The imaging parameters for scans conducted prior to the upgrade were as follows: The imaging parameters for the Trio (12 channel head coil) system consisted of a 6 min high-resolution T1 weighted magnetization-prepared rapid gradient echo (MP-RAGE) scan (repetition time [TR] = 2250 ms, echo time [TE] = 4.15 ms, 192 slices, 50% slice gap, flip angle = 9, voxel size = 1.0 mm3, Field of View [FOV] = 256 mm2, iPAT factor of 2, and using a sagittal, ascending acquisition). The imaging parameters for the Prisma (20 channel head coil) system included an acquisition of 6-min high-resolution T1 weighted MP-RAGE scan (same parameters as Trio, except that TE = 4.11 ms). All participants were scanned at baseline pain level (inter-ictal migraine phase). Any patients reporting acute migraine within the previous 24 h were rescheduled.

### MRI preprocessing

#### Calculation of cortical thickness and subcortical volume

Cortical reconstruction and volumetric segmentation were performed with the FreeSurfer image analysis suite, which is documented and freely available for download online (http://surfer.nmr.mgh.harvard.edu/). The technical details of these procedures are described in prior publications [26–37].

All data were put through a standard processing pipeline which included motion correction and averaging [36] of the T1-weighted scan described above, removal of non-brain tissue using a hybrid watershed/surface deformation procedure [29, 36], automated Talairach transformation, segmentation of the subcortical white matter and deep gray matter volumetric structures (including hippocampus, amygdala, caudate, putamen, ventricles) [28, 29] intensity normalisation [38], tessellation of the gray matter white matter boundary, automated topology correction [39, 40], and surface deformation following intensity gradients to optimally place the gray/white and gray/cerebrospinal fluid borders at the location where the greatest shift in intensity defines the transition to the other tissue class [26, 27, 41].

Because our study acquired from the same person at multiple timepoints we used the FreeSurfer longitudinal pipeline in which an unbiased within-subject template image [42, 43] is created using robust, inverse consistent registration [36]. Several processing steps, such as skull stripping, Talairach transforms, atlas registration as well as spherical surface maps and parcellations were then initialized with common information from the within-subject template, significantly increasing reliability and statistical power [36, 37].Once the cortical models were estimated, cortical thickness was calculated for each region generated by a parcellation of the cerebral cortex into units with respect to gyral and sulcal structure [32, 44]. Data from the cortical regions demarcated by the Destrieux atlas were used in additional analyses described below [45]. Specifically, grey matter was segmented into 148 separate regions demarcated by Destrieux [45] while subcortical structures were segmented into 40 distinct regions based upon probabilistic mapping studies of subcortical structure location [28]. Procedures for the measurement of cortical thickness have been validated against histological analysis [46] and manual measurements [47, 48]. FreeSurfer morphometric procedures have been demonstrated to show good test-retest reliability across scanner manufacturers and across field strengths [33, 37].

### Statistical analysis

Differences in volume and thickness, of subcortical and cortical sites respectively, generated by FreeSurfer were used as the primary dependent variables in our targeted statistical analyses. As suggested by Westman and colleagues [45, 49], the volume of subcortical structures was normalized by the estimated total intracranial volume (eTIV), while cortical thickness values were not normalized. In order to isolate significant changes between the pre-treatment and post-treatment measurements within our a priori regions of interest we conducted a series of two-tailed, paired samples t-tests.

## Results

### Behavioral and clinical data

Pre and post-treatment clinical data were compared using a series of one-tailed (as we hypothesized all measures would show improvement) paired t-tests. Compared to baseline values, the number of moderate/severe headache days per month (*p* < .001), HIT-6 score (*p* < .005), PHQ-9 scores (*p* < .01) and allodynia scores (*p* < .001) were all significantly improved at the end of treatment. All behavioral data are summarized in Table 1.

**Table 1:** Demographic and clinical features of chronic migraine patients pre- and post-treatment

**Demographics**		
Sample Size (n)	12	
Age (Years)	44.3 ± 12.2	
Gender	12 female	
Race/Ethnicity	9 white / 3 black	
**Clinical Measures**		
History of Migraine (Years)	22.3 ± 13.2	
History of Chronic Migraine (Years)	2.1 ± 1.2	
Cranial Autonomic Symptoms:	Yes = 8 / No = 4	
**Measure**	**Baseline**	**End of Treatment**
***Moderate to severe HA (days/month)	20.7 ± 7.2	9.8 ± 7.2
**Allodynia Score	5.7 ± 3.1	2 ± 2.5
***HIT-6 (Headache Impact Test)	65.7 ± 2.9	57.9 ± 4.9
*PHQ-9 (Patient Health Questionnaire)	9.9 ± 6.6	4.3 ± 2.8

Significance of pre-post difference indicated by asterisks (*** *p* < 0.0005, ** *p* < 0.005, * *p* < 0.05). *Abbreviations*: *BMI* Body mass index, *HA* Moderate to severe headache days, *ASC-12* Allodynia symptoms checklist, *HIT-6* Headache impact test, *PHQ-9* Patient health questionnaire-9

### MRI data

#### Subcortical volume differences pre vs post

**Fig. 1:**
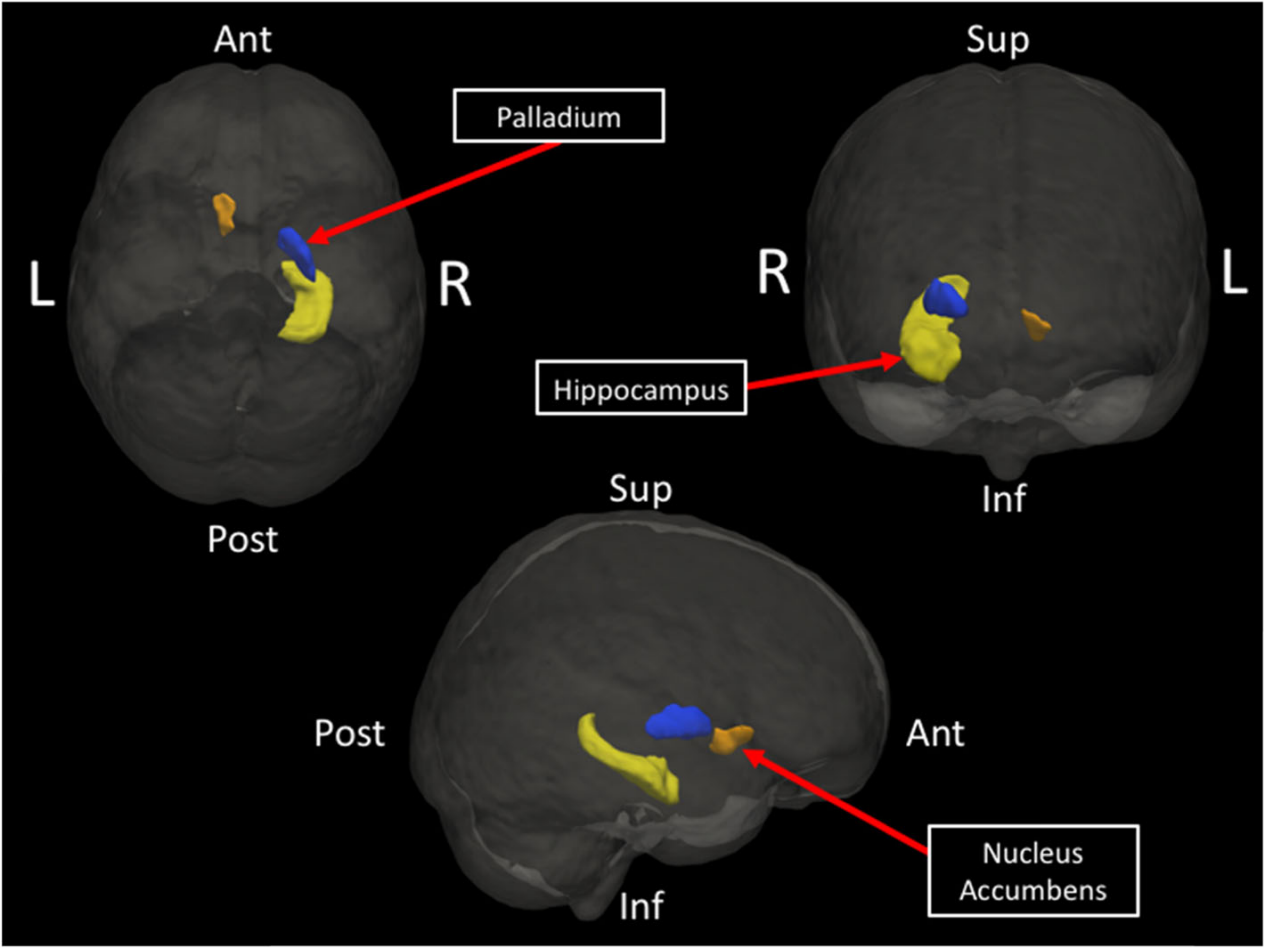
Subcortical structures exhibiting morphometric changes over the course of a 6-week SPG treatment. Volume significantly decreased in the hippocampus (yellow) and palladium (blue), and increased in the nucleus accumbens (orange)

Total volume of subcortical structures recorded at each timepoint was normalized by the estimated total intracranial volume (eTIV) on a participant-by-participant basis to generate a percentage describing the relationship between each structure and the total volume measured. The volume of the right hippocampus, one of our a priori ROIs, significantly decreased following SPG treatment (**M** = 0.315%, **SD** = 0.049) as compared to before treatment (**M** = 0.320%, **SD** = 0.045%), t(11) = − 2.44, *p* < 0.05. Volume of the right palladium also significantly decreased following treatment (**M** = 0.135%, **SD** = 0.04%) as compared to before treatment (**M** = 0.139%, **SD** = 0.028%), t(11) = − 3.06, *p* < 0.05. Volume in the left nucleus accumbens significantly increased following treatment (**M** = 0.037%, **SD** = 0.013%) as compared to before treatment (**M** = 0.032%, **SD** = 0.013%), t(11) = 3.74, *p* < 0.005 (Fig. 1).

#### Cortical thickness differences pre vs post

**Fig. 2:**
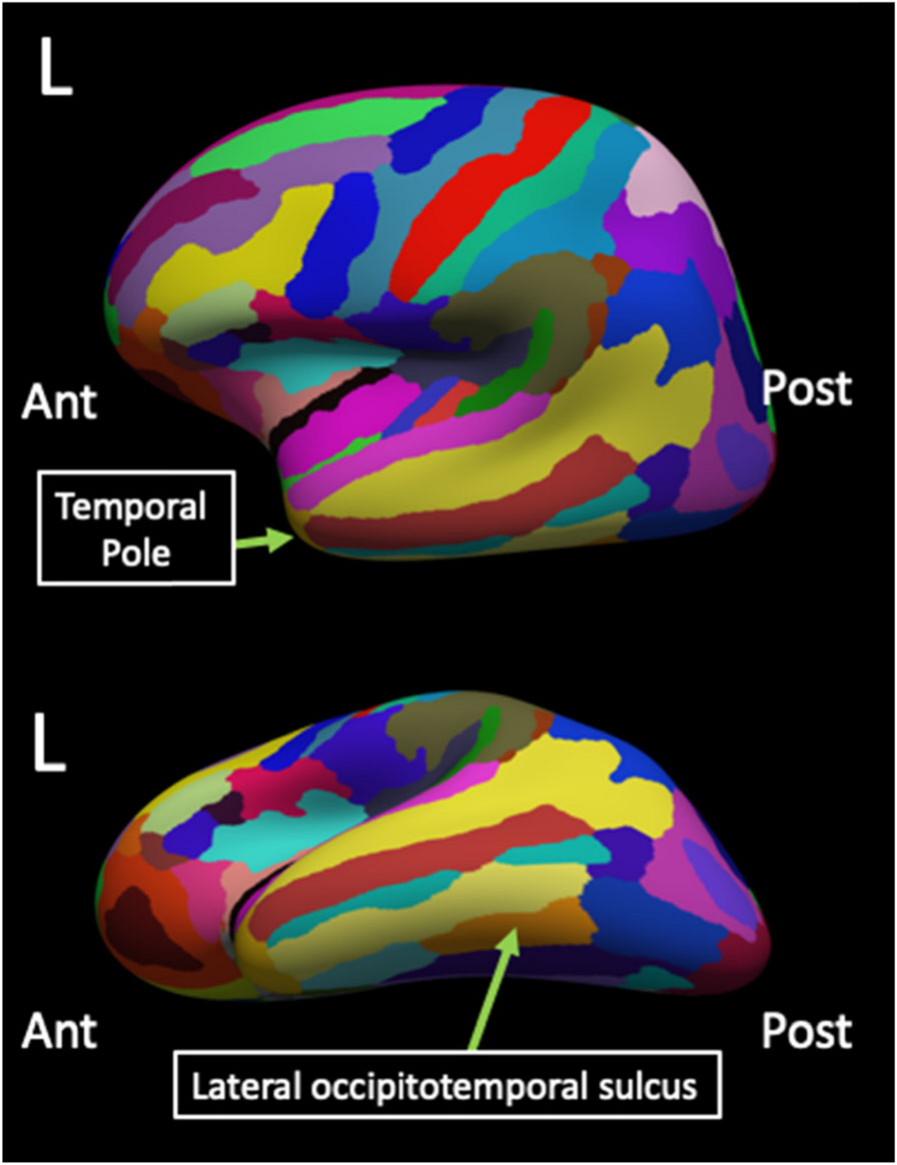
Cortical structures exhibiting morphometric changes after SPG treatment. Over the course of the SPG treatment, cortical thickness decreased in the left temporal pole and the left lateral occipitotemporal sulcus. Ant = anterior, Post = posterior, L = left hemisphere

Cortical thickness within the left temporal pole, one of our a priori ROIs, decreased following treatment (**M** = 3.39 mm, **SD** = 0.16 mm) as compared to before treatment (**M** = 3.44 mm, **SD** = 0.14 mm), t(11) = − 2.22, *p* < 0.05. Cortical thickness within the left lateral occipito-temporal sulcus also decreased following treatment (**M** = 2.49 mm, **SD** = 0.14 mm) relative to before treatment (**M** = 2.55 mm, **SD** = 0.17 mm), t(11) = − 2.50, p < 0.05 (Fig. 2).

## Discussion

The purpose of this paper was to determine the impact of a series of twelve SPG blockades, conducted over a 6-week period, on brain morphology in chronic migraine with medication overuse headaches (CM^w/MOH^). These data represent the first empirical evidence that SPG blockade treatments, such as Tx360 (Tian Medical Inc.; Lombard, IL, USA) are associated with structural changes at cortical and subcortical sites. Importantly, the changes we observed were in areas previously shown to be involved in the migraine pathogenesis. Numerous improvements were reported after the course of the 6-week SPG blockade treatment. The number of moderate/severe headache days per month decreased, the impact of headaches on participants’ ability to function decreased (HIT-6), depression symptomology decreased (PHQ-9) and allodynia decreased (Table 1). These results suggest that the SPG blockade treatment regimen used in the current study had significant beneficial effects.

### Pre-post changes in subcortical volume

#### Hippocampus

Brain imaging investigations into the processing of pain, anxiety and stress support a critical place for the hippocampus in the etiology of migraine [9, 10, 50–52]. More specifically, functional MRI studies report altered resting state connectivity between the hippocampus and other brain regions in migraine [50–52], while structural MRI studies support a positive correlation between headache severity and hippocampal volume in migraine [9, 10]. These findings are consistent with a larger body of literature suggesting that the hippocampus plays a role in the processing of painful stimuli [53–55]. The current study provides novel (in that we measured changes in brain volume) and compelling data that the hippocampus plays an important role in pain processing in chronic migraine with medication overuse headache. Generally speaking, our results mirror the only other study to have investigated the relationship between episodic migraine and hippocampal volume [9]. Critically, while this prior report of structural differences was based on cross sectional data in high and low frequency migraineurs, the present data are based on pre and post treatment data obtained over the course of a relatively short period of time. The fact that measurable changes in hippocampal volume occurred after a series of SPG blockades suggests that the hippocampal region is remarkably mutable and capable of neuroplasticity within the context of chronic migraine treatment.

#### Basal ganglia

In addition to hippocampal changes, we detected morphometric alterations in two additional subcortical areas. Our finding that right palladium volume decreased following SPG treatment is generally consistent with prior research demonstrating a relationship between migraine and basal ganglia structures [16]. In the current study, we observed a relationship between migraine severity and brain volume at a single site within the basal ganglia, namely the right palladium. Our finding of altered volume in the ventral striatum, namely in the nucleus accumbens, is also interesting given prior reports of altered processing in this area in migraine [52, 56], and CM^w/MOH^ [52, 57]. Interestingly, the volume of the nucleus accumbens increased, rather than decreased, following a series of the SPG treatment [58, 59]. Our data clearly demonstrate the involvement of at least two distinct areas within the basal ganglia in migraine. However, other areas within the basal ganglia could still be involved. The failure of the current study to find involvement of other structures may be due to methodological differences, such as inclusion criteria, or the fact that we observed changes occurring within a relatively short period of time (6-weeks).

### Pre-post changes in cortical thickness

Results from numerous prior studies are consistent with a central role of the temporal lobe in the psychophysiology and cyclical recurrence of migraine [19, 60–64]. For example, painful heat has been shown to elicit greater temporal pole (TP) activation in migraineurs as compared to healthy controls [12]. Additionally, Schwedt and colleagues reported that TP volume was a key component of classifiers able to distinguish high and low frequency migraineurs [65]. In the current study, we report decreased cortical thickness within the left temporal pole following SPG treatment, showing incomplete normalization as compared to HC. While decreases in excitability and volume are not necessarily the same, it has been argued that the growth of specific brain areas may be related to the cognitive demands of the environment [66, 67] perhaps due to changes in localized neuronal spine density [68]. Given this argument, our results appear to be consistent with those of Moulton and colleagues [12] as well as the larger body of evidence implicating the temporal lobe in migraine etiology.

In addition to the temporal pole we also report a significant decrease in cortical thickness within the lateral occipito-temporal sulcus. This area is located on the inferior aspect of the temporal lobe and is bounded by the inferior occipital gyrus posteriorly and the temporal pole anterior, and roughly corresponds to the visual processing area referred to as the MT/V5 (middle temporal visual) complex. At least one other study has reported abnormalities localized to this area in migraine [69, 70]. In both studies, cortical thickness in the same left occipito-temporal area was found to be relatively increased in patients with migraine as compared to a healthy cohort [70]. Our data provide further evidence that this area is involved in migraine pathophysiology and capable of significant neuroplastic repair. While the exact role of this area in migraine pathophysiology has not yet been confirmed, it may be responsible for impaired visual processing among migraineurs during and between attacks [71]. It is likely that deleterious neuroplastic changes associated with high frequency migraine, specifically within the lateral occipito-temporal sulcus, are reversible given the adoption of an effective treatment regime.

## Conclusion

In this study, we investigated changes in brain morphology following a series of SPG treatments in CM^w/MOH^. We found significant post-intervention changes in both the volume of subcortical structures and the thickness of cortical structures implicated in migraine pathophysiology. Despite some of the limitations, we present novel evidence of neuroplasticity associated with SPG treatment in CM^wMOH^, highlighting the critical roles of previously known cortical and subcortical structures involved in migraine pathophysiology. Most importantly, our data demonstrate that these areas are malleable (our brain has the capacity to change as chronic migraine improves). An improved understanding of how the brain changes as CM^wMOH^ develops and improves has the potential to inform the development of new therapeutic targets, as well as clinical management of existing CM^wMOH^. Future studies should use larger samples to examine morphometric brain changes associated with other types of headaches, as well as potential changes associated with SPG blockade and alternative treatments.

### Limitations

The results of this study must be interpreted with caution given numerous limitations and potential confounds. The biggest limitation of this study is the small sample size. One reason we were able to find significant differences is likely our adoption of a within-subjects design that minimized the influence of inter-subject variability on our statistical tests. It is worth noting that we observed a significant decrease in pain medication usage across the study, which occurred in all of our patients, and is likely due to reduced headache frequency. Furthermore, our study does not comment on whether the brain morphology changes we observed are the result of SPG blockade per se as opposed to the reduced frequency of migraine or general reduction in chronic pain observed in our participants. While we have used the conventional terms to refer to volume/thickness increases and decreases (which have proved reliable in long term longitudinal studies), caution is advised regarding the mechanism of these changes (i.e. we do not know whether these changes are due to changes in white matter fiber tract or glial cells). This is a common limitation for MRI analysis using FreeSurfer. Another concern is that, in addition to the SPG, our treatment application may have affected other branches of the trigeminal nerve, such as the maxillary branch. We also note that, while we did collect MRI data on two different MRI systems, it was never the case that pre and post measurements were made on different MRI systems. However, it is possible that our results were affected by our use of two different scanners over the course of the experiment (i.e. results may have been better using one scanner configuration as opposed to the other). Finally, because all of our participants were female, and it is known that changes in brain volume, particularly in the hippocampus, may be related to the menstrual cycle [72], we cannot rule out the possibility that changes observed in the current study were due to this uncontrolled variable. Due to the time course of a series of SPG treatment (6 weeks), it is not feasible to control this potential confounder.

## Data Availability

The datasets used and analyzed during the current study are available from the corresponding author on reasonable request.
